# A New Model of Diarrhea with Spleen-Kidney Yang Deficiency Syndrome

**DOI:** 10.1155/2018/4280343

**Published:** 2018-09-30

**Authors:** Jiajie Zhu, Shan Liu, Yu Guo, Liwei Hou, Xiaolan Su, Yijie Li, Boyu Han, Dengke Liu, Qingguo Wang, Jiande JD Chen, Wei Wei

**Affiliations:** ^1^Department of Gastroenterology, Tongde Hospital of Zhejiang Province, Hangzhou 310012, China; ^2^Department of Gastroenterology, Wangjing Hospital, China Academy of Chinese Medical Sciences, Beijing 100102, China; ^3^Dongzhimen Hospital, Beijing University of Chinese Medicine, Beijing 100700, China; ^4^Basic Medical College, Zhejiang Chinese Medical University, Hangzhou 310053, China; ^5^Traditional Chinese Medicine Department of Gastroenterology, China-Japan Friendship Hospital, Beijing 100029, China; ^6^College of Traditional Chinese Medicine, Beijing University of Chinese Medicine, Beijing 100029, China; ^7^Division of Gastroenterology and Hepatology, Johns Hopkins University, School of Medicine, Baltimore, MD 21224, USA

## Abstract

**Objective:**

The aim of this study was to examine a new method to create a rat model of diarrhea with spleen-kidney yang deficiency syndrome.

**Methods:**

A senna leaf (Folium sennae) decoction was made in 3 concentrations of 1.0, 0.5, and 0.25 g/mL. Rats were randomly divided into 4 groups: the control (C)-, high (H)-, middle (M)-, and low (L)- dose groups. The groups received saline, 1.0, 0.5, or 0.25 g/mL senna leaf decoction, respectively, for 4 weeks. Body weight monitoring, food consumption, water intake, defecation frequency, stool Bristol score, weight-loaded forced swimming test, forelimb grip strength test, D-xylose absorption test, serum cortisone, adrenocorticotropic hormone (ACTH), 24 h urine 17-hydroxycorticosteroid (17-OHCS), and histopathological detection were conducted to assess the success of the senna leaf decoction-induced model.

**Results:**

This study showed that the senna leaf decoction could induce diarrhea and dose-dependently slow body weight growth, reduce food consumption, and increase water intake, stool Bristol score, and defecation frequency. Statistical differences were found between groups H and M in rectal temperature, weight-loaded forced swimming time, forelimb grip strength, and serum cortisone. The D-xylose absorption test also showed dysfunction of intestinal absorption in groups H and M. The serum cortisone and 24 h urine 17-OHCS were significantly reduced in group H.

**Conclusions:**

Gastric gavage of 10 mL/kg of body weight of a high concentration of a senna leaf decoction (1.0 g/mL) for 4 weeks was used to create a rat model of diarrhea with spleen-kidney yang deficiency syndrome.

## 1. Introduction

Diarrhea, characterized as increased stool frequency and loose or watery fecal consistency, is one of the most common gastrointestinal disorders in clinic. It is often classified as acute or chronic. Acute diarrhea lasts from a few days to a week; chronic diarrhea can be defined in several ways, but most types last more than three weeks [1]. Obviously, functional diarrhea (FDr) is categorized to the latter according to the Rome IV criteria [2]. With the prevalence of 1.5%–17% [3–7], FDr substantially affected the quality of life, especially in role-physical, general health, vitality, social functioning, and role-emotional domains of the 36-item Short Form health survey (SF-36) [5].

In traditional Chinese medicine (TCM) theory, the spleen controls transportation and transformation, while the kidney yang warms the spleen to insure its function. Yang deficiency in the spleen or kidney would cause dysfunction of transportation and transformation, thus leading to diarrhea [8]. In clinical cases, spleen-kidney yang deficiency syndrome is one of the most common syndromes of FDr, especially in patients with a long history [9].

Invigorating the spleen and warming the kidney are two of the classical nine methods of treating diarrhea, which were firstly documented in Yizong Bidu (written by ZhongZi Li in the Ming dynasty). With a satisfied efficacy, the two treating methods were widely used, alone or combined, in treating chronic diarrhea [10]. However, the mechanisms of invigorating the spleen and warming the kidney are still poorly understood. To further investigate those mechanisms, a diarrhea model with the characteristics of spleen-kidney yang deficiency syndrome is required. However, few studies have examined this model.

Senna leaf (Folium Sennae), which contains anthraquinone glycoside, like sennoside [11], as the main effective component, has a strong effect of laxative [12]. Therefore, it has been widely used for treating constipation and bowel preparation in clinic and has a good tolerability even in colon cancer patients [13, 14]. In addition, it is commonly used to create diarrhea models in animal studies [15].

However, according to TCM theory, this herb is cold in property and bitter in flavor (Chinese Pharmacopeia 2010 version). Short-term use is tolerable, but excessive consumption or improper use could cause diarrhea and impairment of the spleen yang [16], and long-term use would involve the kidney yang, ultimately leading to spleen-kidney yang deficiency syndrome. In a study by Chen and Wei [16], Senna Leaf was used at 0.8 g dose for 12 days to build a rat model of diarrhea with spleen yang deficiency syndrome. Therefore, we hypothesized that use of senna leaf for 4 weeks would create a rat model of diarrhea with spleen-kidney yang deficiency syndrome.

## 2. Material and Method

### 2.1. Herbal Preparation

Senna leaf was purchased from Wangjing Hospital, China Academy of Chinese Medical Science (CACMS). Each 100 g of senna leaf was soaked in 400 mL boiled distilled water for 30 min, boiled for 3 min, and filtered through two-tier gauze. The filtrate was concentrated or diluted to three concentrations, 1.0 g/mL, 0.5 g/mL, and 0.25 g/mL, and stored at 4°C.

### 2.2. Experimental Animals

Twenty-four male Wistar rats (70 ± 10 g) were obtained from the Laboratory Animal Center of the Academy of Military Medical Sciences (China). The animals were housed at the Animal Center of the Institute of Basic Theory for Chinese Medicine, CACMS, under a 12/12 light cycle with standard temperature (21–23°C) and humidity (50% * *± 5%), and provided with food and water ad libitum. All the experimental protocols were approved by the Animal Ethics Committee of the CACMS.

### 2.3. Experimental Design

After 5 days of acclimatization, rats were randomly divided into 4 groups of 6 rats each. The control group (C) received saline. The high dose (H), middle dose (M), and low dose (L) groups received 1.0, 0.5, or 0.25 g/mL senna leaf solution, respectively. All solutions were administered through gavage at 10 mL/kg of body weight, once per day, for 4 weeks.

### 2.4. Behavioral Observation

The body weight and rectal temperature of the rats were recorded weekly. Weighed food and water were added to the containers of each cage, and the residual food and water were measured daily.

Rats were housed in individual cages with metal grid bottoms. A piece of filter paper was placed under each cage to collect feces. After 4 hours, fecal pellets were counted and the stool form was assessed using the Bristol scale [2].

The weight-loaded forced swimming test (WLFST) was used according to Qi's method [17] with some modifications. Each rat swam with a load of lead rings that weighed approximately 10% of its body weight and was attached to its tail. The swimming time was measured from the beginning until the point at which the rat could not return to the surface of the water 8 s after sinking. Then, rats were helped out of the tank and returned to their cages for recovery.

The forelimb grip strength test (FGST) was conducted using a force testing system (model-YLS-13A, ZS Dichuang Ltd., Beijing, China). The maximum grip strength was recorded when the rat grasped the metal bar of the equipment and was steadily pulled away until the rat released the bar [18]. Each rat performed the FGST three times, with an interval of 10 minutes between each test.

### 2.5. Sample Collection and Hormone Level Measurements

Urine samples were collected weekly from metabolic cages and centrifuged 10 minutes at 2500 rpm and 4°C (TGL-20MS, Lu Xiangyi Centrifuge Instrument Co., Ltd., Shanghai, China). Then the supernatants were collected. After 4 weeks, rats were given a 3% D-xylose solution by gavage at 10 mL/kg of body weight, except 2 in group C, after 18 hours' fasting. Then the rats were anesthetized with 10% chloral hydrate through intraperitoneal injection at 3 mL/kg of body weight. Blood samples were obtained from the abdominal aorta and centrifuged for 15 minutes at 3000 rpm and 4°C, and then the serum was collected. All samples were stored at −80°C until biochemical analysis.

The serum cortisone, adrenocorticotropic hormone (ACTH), and 24 h urine 17-hydroxycorticosteroid (17-OHCS) levels were measured using ELISA kits (EIAaB Science Co., Ltd., Wuhan, China). The serum D-xylose level was measured with a D-xylose assay kit (Megazyme Ltd., Wicklow, Ireland).

### 2.6. HE Staining Process

The rats were decapitated, and a 1 cm colon (7 cm above the anus) was taken out and cleaned with a phosphate buffer solution. Each colon segment was fixed in 10% formalin, routinely dehydrated, embedded into paraffin, continuously sliced, deparaffinized, and rehydrated. Staining was performed using the hematoxylin and eosin stain. Then the hematoxylin was dehydrated in 70%, 90%, and 95% ethanol, cleared in xylene, and mounted in Permount or Histoclad. Morphological changes were observed under a microscope.

### 2.7. Data Processing and Statistical Analysis

Calculations were performed in Excel (Microsoft Corp., Redmond, WA, USA) and statistical analyses were performed using SPSS Statistics for Windows (Version 20.0, IBM Corp. Armonk, NY, USA). Normally distributed data were expressed as mean value ± standard deviation (SD) and analyzed using a one-way analysis of variance (ANOVA). The one-way nonparametric ANOVA Kruskal-Wallis test was used to analyze nonnormally distributed data. Least significant difference (LSD) tests were performed for post hoc multiple treatment comparisons. A *P* value < 0.05 was considered significant. All *P* values were reported till 3 decimals and the zero *P* value was used in form of less than 0.001.

## 3. Result

### 3.1. Behavioral Presentation

During model construction, the rats in group C were active, had neat and lustrous fur, and produced granular fecal pellets. However, the rats in group H had diarrhea and poor appetite from initial administration of the senna leaf solution. They gradually presented with signs of exhaustion, such as reduced activity, idleness, slow response, tendency to cluster, and depilation. These symptoms were in accordance with those of patients with spleen-kidney yang deficiency. Rats in groups M and L had similar changes to a lesser degree.

**Figure 1:**
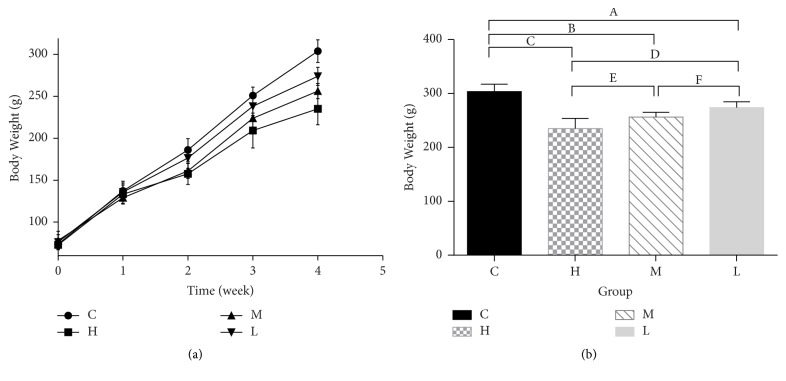
Body weight of the four groups. (a) Line graph of the body weights of rats during the 4 weeks. (b) Column graph of the body weights of rats at the end of the 4th week. ^A^*P* = 0.001, ^B,C^*P* = 0, ^D^*P* = 0, ^E^*P* = 0.014, and ^F^*P* = 0.037. C, control group. H, high dose group. M, middle dose group. L, low dose group.

The body weights of the rats are provided in Figure 1 and Table S1 (see supplementary material). After the second week of gavage, the weights of groups H and M were significantly lower than group C (157.6 ± 12.7, 161.9 ± 8.8 versus 186.0 ± 13.4 g, *P* = 0.001 and *P* = 0.002) and group L (157.6 ± 12.7, 161.9 ± 8.8 versus176.5 ± 12.2 g, *P* = 0.013 and 0.047). At the end of the fourth week, body weight and senna leaf solution presented a dose-dependent relationship: the body weight of rats in group H was significantly lower than that of groups C, L (235.0 ± 19.0 versus 303.8 ± 13.4, 273.8 ± 10.8 g, *P* = 0 and 0), and M (235.0 ± 19.0 versus 256.2 ± 9.0 g, *P* = 0.014). The weight of rats in group M was significantly lower than that of groups C and L (256.2 ± 9.0 versus 303.8 ± 13.4, 273.8 ± 10.8 g, *P* = 0 and 0.037). The weight of rats in group L was significantly lower than that of group C (273.8 ± 10.8 versus 303.8 ± 13.4 g, *P* = 0.001).

**Figure 2:**
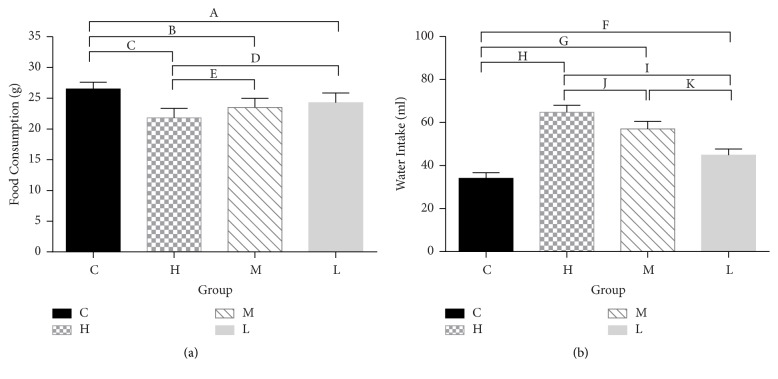
Food consumption and water intake. (a) The food consumption of the rats. (b) The water intakes of the rats. ^A^*P* = 0.008, ^B^*P* = 0.001, ^C^*P* = 0, ^D^*P* = 0.003, ^E^*P* = 0.037, ^F,G,H,I,J,K^*P* = 0. C, control group. H, high dose group. M, middle dose group. L, low dose group.

The food consumption and water intake of each group after 4 weeks of oral administration of senna leaf solution are shown in Figure 2 and Table S2 (see supplementary material). Although all groups showed reduced food consumption and increased water intake compared to group C (21.79 ± 1.55, 23.48 ± 1.49 and 24.29 ± 1.56 versus 26.51 ± 1.09 g/d, *P* = 0, 0.001 and 0.008), there were also differences among the 3 groups. Group H showed decreased food consumption compared with groups M (21.79 ± 1.55 versus 23.48 ± 1.49 g/d, *P* = 0.037) and L (21.79 ± 1.55 versus 24.29 ± 1.56 g/d, *P* = 0.003). In addition, the effect of the senna leaf solution on water intake increase was dose-dependent (Figure 2).

**Figure 3:**
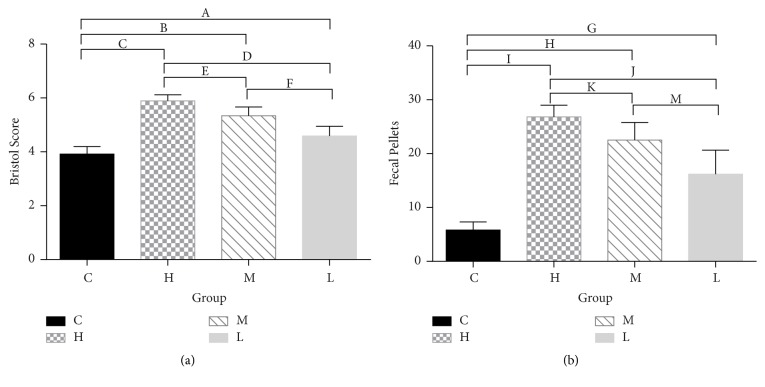
Fecal grains and Bristol scores. (a) The average Bristol score of each group. (b) The average fecal grain in 4 hours of each group. ^A,B,C,D^*P* = 0, ^E^*P* = 0.005, ^F^*P* = 0.002, ^G,H,I,J,M^*P* = 0, ^K^*P* = 0.023. C, control group. H, high dose group. M, middle dose group. L, low dose group.

After 4 weeks of administration, the rats in group C defecated granular feces that did not stain filter paper, while rats in the groups that received the senna leaf solution had much more frequent bowel movements and higher Bristol scores (*P* < 0.01) (Figure 3) (Table S3 see supplementary material).

The changes in rectal temperature are shown in Table 1. After 3 weeks of senna leaf administration, the rectal temperature of group H was significantly lower than that of group C (38.49 ± 0.24 versus 38.86 ± 0.23°C, *P* = 0.022). Until the end of model construction, the temperature of group H was lower than that of groups C (38.48 ± 0.19 versus 38.96 ± 0.27°C, *P* = 0.004) and L (38.48 ± 0.19 versus 38.84 ± 0.27°C, *P* = 0.024). The temperature of group M was also lower than that of group C (38.62 ± 0.29 versus 38.96 ± 0.27°C, *P* = 0.031).

**Figure 4:**
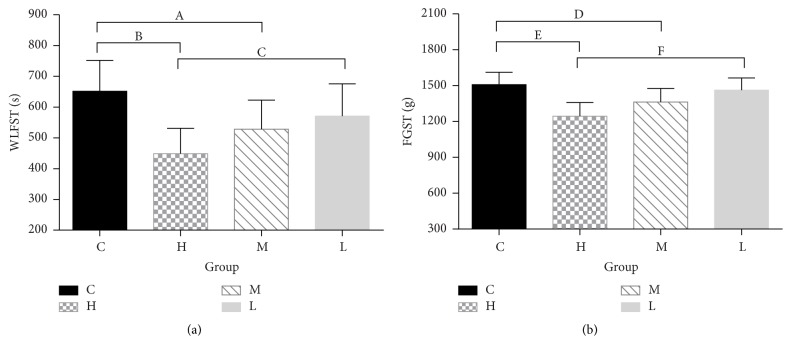
Physical activities of the rats. (a) WLFST of each group. (b) FGST of each group. ^A^*P* = 0.037, ^B^*P* = 0.002, ^C^*P* = 0.039, ^D^*P* = 0.032, ^E^*P* = 0, and ^F^*P* = 0.002. WLFST, weight-loaded forced swimming test. FGST, forelimb grip strength test. C, control group. H, high dose group. M, middle dose group. L, low dose group.

When comparing physical activities, the WLFST and FGST had similar results. At the end of the fourth week, the swimming time and grip strength of group H were significantly lower than those of groups C (WLFST: 448.66 ± 82.67 versus 651.83 ± 100.19 s, *P* = 0.002; FGST: 1244.53 ± 113.53 versus 1507.92 ± 103.94 g, *P* = 0) and L (WLFST: 448.66 ± 82.67 versus 570.75 ± 105.12 s, *P* = 0.039; FGST: 1244.53 ± 113.53 versus 1462.17 ± 102.27 g, *P* = 0.002). Additionally, the swimming time and grip strength of group M were also lower than those of group C (WLFST: 528.27 ± 94.52 versus 651.83 ± 100.19, *P* = 0.037; FGST: 1362.76 ± 114.66 versus 1507.92 ± 103.94 g, *P* = 0.032) (Figure 4) (Table S4 see supplementary material).

### 3.2. Serum Hormone and D-Xylose Level Measurements

**Figure 5:**
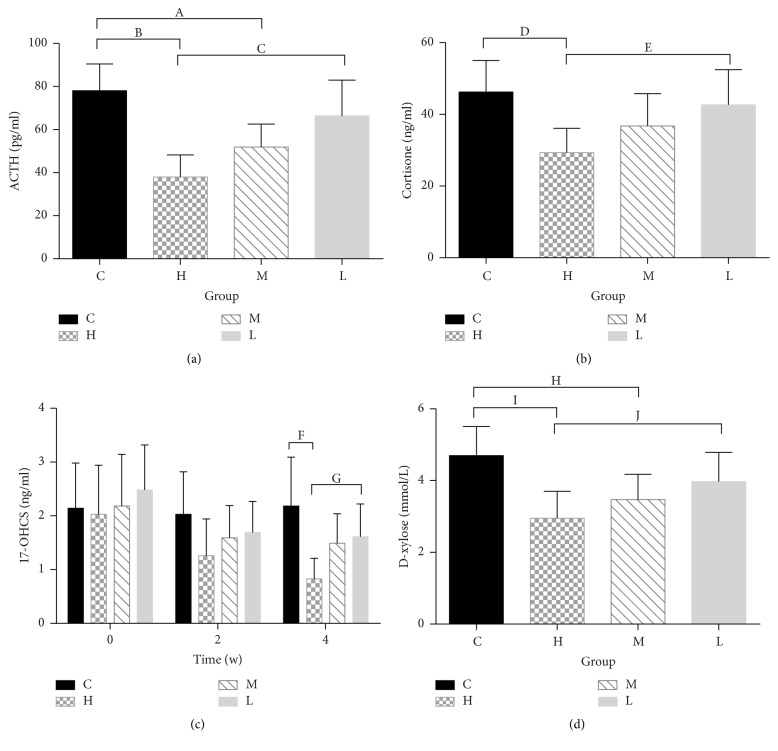
Hormone level and serum D-xylose content. (a) ACTH of each group. (b) Cortisone of each group. (c) 17-OHCS of each group during the 4 weeks. (d) D-xylose of each group. ^A^*P* = 0.002, ^B^*P* = 0, ^C^*P* = 0.001, ^D^*P* = 0.003, ^E^*P* = 0.016, ^F^*P* = 0.002, ^G^*P* = 0.047, ^H^*P* = 0.012, ^I^*P* = 0.001, and ^J^*P* = 0.033. ACTH, adrenocorticotropic hormone. 17-OHCS, 17-hydroxycorticosteroids. C, control group. H, high dose group. M, middle dose group. L, low dose group.

The serum hormone and D-xylose level are provided in Figure 5 and Table S5 (see supplementary material). For 17-OHCS and cortisone, group H showed significant reductions compared with group C (17-OHCS: 0.83 ± 0.38 versus 2.18 ± 0.91 ng/ml, *P* = 0.002; cortisone: 29.34 ± 6.79 versus 46.21 ± 8.83 ng/ml, *P* = 0.003) and group L (17-OHCS: 0.83 ± 0.38 versus 1.61 ± 0.61 ng/ml, *P* = 0.047; cortisone: 29.34 ± 6.79 versus 42.65 ± 9.84 ng/ml, *P* = 0.016) after 4 weeks of administration. While serum ACTH was more sensitive, the levels in groups H and M were conspicuously lower than that in group C (37.97 ± 10.21, 51.91 ± 10.59 versus 78.03 ± 12.39 ng/ml, *P* = 0 and 0.002). Meanwhile, group H also had a lower level than group L (37.97 ± 10.21 versus 66.34 ± 16.61 ng/ml, *P* = 0.001). Similar results were found in serum D-xylose levels.

### 3.3. Intestinal HE Staining

**Figure 6:**
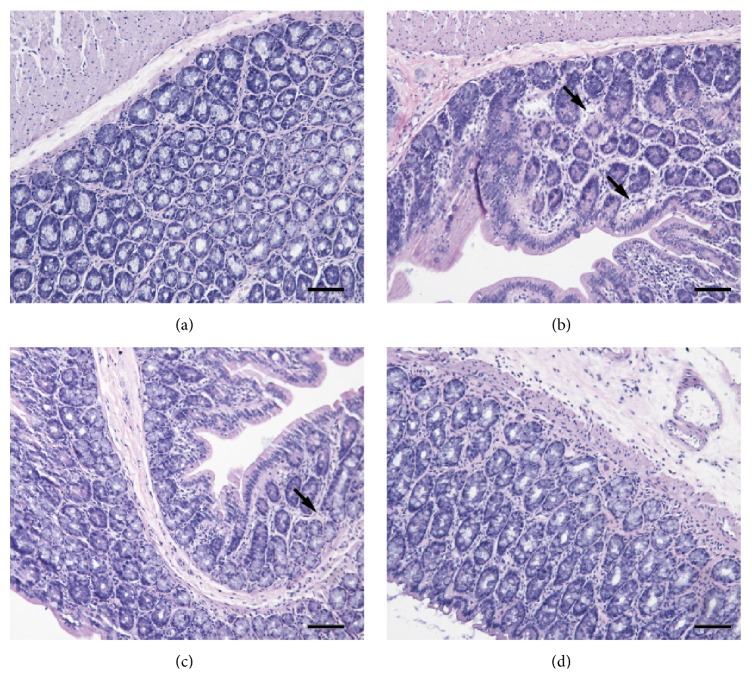
Intestinal HE dyeing. (a) Control group. (b) High dose group. (c) Middle dose group. (d) Low dose group. Each group had complete colon structure and aligned cells, and there was no evident abnormal changes, such as erosion or ulcer. Mild swellings were pointed out by arrowheads. Bar = 100 um.

Rat colon HE staining results are shown in Figure 6. Each group had a complete colon structure and aligned cells, and there were no evident abnormal changes, such as erosion or ulcer. In groups H and M, mild swellings were visible under the mucous membrane.

## 4. Discussion

### 4.1. Main Findings

In TCM, the normal function of the spleen aids digestion; spleen yang deficiency disturbs this process, leading to poor digestion and weight loss [19, 20]. The D-xylose absorption test assesses the intestinal capacity to absorb the simple sugar D-xylose as an indicator of adequate nutrient absorption [21] and is commonly used to indicate the condition of spleen yang deficiency [22]. We applied this test with body weight monitoring, food, and water intake measurements to evaluate the success of senna leaf-induced spleen yang deficiency.

TCM also posits that the kidney yang, the root of the yang qi, is the motivity of physical activity and has a warming effect on the body. Declines in physical activity and body temperature have been regarded as characteristics of kidney yang deficiency syndrome [23, 24]. WLFST and FGST, measurements to assess physical activities [17, 25], serum cortisone, ACTH, and 24 h urine 17-OHCS, which were utilized to assess kidney yang deficiency in TCM [26], as well as rectal temperature measurement, were conducted to determine the success of senna leaf-induced kidney yang deficiency.

This study showed that a senna leaf solution could induce diarrhea and dose-dependently slow weight gain, reduce food consumption, and increase water intake. The D-xylose absorption test also showed dysfunction of intestinal absorption in the groups that received the high and middle dose senna leaf solutions. For the WLFST, FGST, rectal temperature, and serum cortisone, statistical decreases were also observed in groups H and M. Serum ACTH and 24 h urine 17-OHCS were significantly reduced in group H only. Based on the above information, we could conclude that gavage with a high dose senna leaf solution (1.0 g/mL; 10 mL/kg) for 4 weeks was the acceptable approach to create a rat model of diarrhea with spleen-kidney yang deficiency syndrome.

### 4.2. Interpretations

Rhubarb* (Radix et Rhizoma Rhei)* and senna leaf are the most well-known laxatives in TCM. The main purgatives in these crude drugs are the sennosides, including sennoside A and sennoside B [11]. Their laxative effect was proved by decreasing the expression of aquaporin-3 (AQP3) in colon mucosal epithelial cells, which inhibited water absorption from the intestinal tract [27], increasing the rate of colonic motility and enhancing colonic transit [14], leading to diarrhea.

However, according to TCM, herbs with a cold property and a bitter flavor could impair the spleen yang and result in dysfunction of transportation and transformation, inducing diarrhea. On the basis of this theory, these herbs were commonly used to create animal models with spleen deficiency syndrome in TCM researches [16, 20, 28]. Additionally, excessive use of these herbs could not only impair the spleen yang, but also involve the kidney yang in a long-term case, eventually causing spleen-kidney yang deficiency syndrome [29].

### 4.3. Limitations

Animal models with TCM syndromes are the basis of TCM research. An increased number of TCM syndrome models have recently been established for use in TCM studies [30]. The evaluation criteria of a model are crucial. Four-dimensional criteria including behavioral observation, relative biomarkers, drug counterevidence, and conjecture according to the creation factors have been proposed in China [31]. The current study aimed to determine the suitable dosage of senna leaf solution to create a rat model of diarrhea with spleen-kidney yang deficiency syndrome. No attempt was made to apply drug counterevidence in the current study because there were 3 model groups. Once we determine the optimal dosage for the model, drug counterevidence will be applied to enhance the method.

## 5. Conclusion

Our study revealed that oral administration of a senna leaf solution is a new method to create an animal model of diarrhea with spleen-kidney yang deficiency syndrome.

## Figures and Tables

**Table 1:** Rectal temperature of the four groups.

Group	*N*	0 w	1 w	2 w	3 w	4 w
C	6	37.97 ± 0.24	38.54 ± 0.27	38.70 ± 0.28	38.86 ± 0.23	38.96 ± 0.27
H	6	37.99 ± 0.17	38.33 ± 0.33	38.40 ± 0.15	38.49 ± 0.24^a^	38.48 ± 0.19^bc^
M	6	37.97 ± 0.23	38.52 ± 0.27	38.59 ± 0.28	38.62 ± 0.23	38.62 ± 0.29^d^
L	6	38.02 ± 0.20	38.51 ± 0.19	38.64 ± 0.29	38.69 ± 0.32	38.84 ± 0.27

^a^ *P* = 0.022, ^b^*P* = 0.004, and ^d^*P* = 0.031, compared with group C. ^c^*P* = 0.024, compared with group L. C, control group. H, high dose group. M, middle dose group. L, low dose group.

## Supplementary Materials

Supplementary Materials Supplementary Material Supplementary Table 1: body weight of the four groups. Supplementary Table 2: food consumption and water intake of the four groups. Supplementary Table 3: diarrhea of the four groups. Supplementary Table 4: physical activities of the four groups. Supplementary Table 5: hormone level and serum D-xylose content of the four groups.

## References

[1] Wang S., Zhao Y., Zhang J. (2015). Antidiarrheal effect of Alpinia oxyphylla Miq. (Zingiberaceae) in experimental mice and its possible mechanism of action. *Journal of Ethnopharmacology*.

[2] Lacy B. E., Mearin F., Chang L. (2016). Bowel disorders. *Gastroenterology*.

[3] Adane M., Mengistie B., Kloos H., Medhin G., Mulat W. (2017). Sanitation facilities, hygienic conditions, and prevalence of acute diarrhea among underfive children in slums of Addis Ababa, Ethiopia: Baseline survey of a longitudinal study. *PLoS ONE*.

[4] Schmulson M., Ortíz O., Santiago-Lomeli M. (2006). Frequency of functional bowel disorders among healthy volunteers in Mexico City. *Digestive Diseases*.

[5] Zhao Y.-F., Guo X.-J., Zhang Z.-S. (2012). Epidemiology of functional diarrhea and comparison with diarrhea-predominant irritable bowel syndrome: a population-based survey in China. *PLoS ONE*.

[6] Chang F. Y., Chen P. H., Wu T. C. (2012). Prevalence of functional gastrointestinal disorders in Taiwan: questionnaire-based survey for adults based on the Rome III criteria. *Asia Pacific Journal of Clinical Nutrition*.

[7] Sorouri M., Pourhoseingholi M. A., Vahedi M. (2010). Functional bowel disorders in Iranian population using Rome III criteria. *Saudi Journal of Gastroenterology*.

[8] Liu Y., Bai S. J., Ma J. (2015). Research progress of functional diarrhea treated with Chinese medicine. *Journal of Liaoning University of Traditional Chinese Medicine*.

[9] Lan X. Y., Deng N., Li X., Guo Z., Yi Z., Zheng J. (2016). *Experimental Study of Sishenwan on Model Rats with Diarrhea of Asdthenic Splenonephro-yang*.

[10] Su X. L., Tang Y. P., Zhang J. (2013). Curative effect of warming kidney and fortifying spleen recipe on diarrhea-predominant irritable bowel syndrome. *Journal of Traditional Chinese Medicine*.

[11] Dhanani T., Singh R., Reddy N., Trivedi A., Kumar S. (2017). Comparison on extraction yield of sennoside A and sennoside B from senna (Cassia angustifolia) using conventional and non conventional extraction techniques and their quantification using a validated HPLC-PDA detection method. *Natural Product Research (Formerly Natural Product Letters)*.

[12] Guarize L., Da Costa J. C., Dutra L. B., Mendes R. F., Lima I. V. A., Scio E. (2012). Anti-inflammatory, laxative and intestinal motility effects of Senna macranthera leaves. *Natural Product Research (Formerly Natural Product Letters)*.

[13] Ishibashi K., Kumamoto K., Kuwabara K. (2012). Usefulness of sennoside as an agent for mechanical bowel preparation prior to elective colon cancer surgery. *Asian Journal of Surgery*.

[14] Manukyan M. N., Tolan K., Severge U., Attaallah W., Kebudi A., Cingi A. (2011). Prospective randomized comparison of oral sodium phosphate and sennoside A+B calcium lavage for colonoscopy preparation. *Surgical Laparoscopy Endoscopy & Percutaneous Techniques*.

[15] Wang L., Yao Q., Ye D., Zhang Y., Lin Y. (2016). Effects of rifaximin on visceral sensitivity of rats with diarrhea induced by folium sennae. *International Journal of Clinical and Experimental Medicine*.

[16] Chen D. Z., Wei M. X. (1997). Preliminary study on the pathological model of Piyinxu in rats. *World Journal of Gastroenterology*.

[17] Qi B., Zhang L., Zhang Z., Ouyang J., Huang H. (2014). Effects of ginsenosides-Rb1 on exercise-induced oxidative stress in forced swimming mice. *Pharmacognosy Magazine*.

[18] Chen K., Liu J., Assinck P. (2016). Differential histopathological and behavioral outcomes eight weeks after rat spinal cord injury by contusion, dislocation, and distraction mechanisms. *Journal of Neurotrauma*.

[19] Meng Z. Z., Chen J. X., Jiang Y. M., Zhang H. T. (2013). Effect of xiaoyaosan decoction on learning and memory deficit in rats induced by chronic immobilization stress. *Evidence-Based Complementary and Alternative Medicine*.

[20] Zhang S.-F., Ling C.-Q., Li B., Chen H.-Y., Chen Z. (2012). Effects of sisheng decoction on the immunity and anti-stress function in mice with spleen deficiency syndrome. *Journal of Chinese Integrative Medicine*.

[21] Martínez-Olmos M. A., Peinõ R., Prieto-Tenreiro A. (2013). Intestinal absorption and pancreatic function are preserved in anorexia nervosa patients in both a severely malnourished state and after recovery. *European Eating Disorders Review*.

[22] Wang Y., Wang H., Zheng X. W. (2013). NMR-spectroscopy-based metabonomic study on plasma of rats with spleen-qi defciency pattern and spleen-yang defciency pattern. *China Journal of Traditional Chinese Medicine and Pharmacy*.

[23] Wong Y. C. (2016). Need of integrated dietary therapy for persons with diabetes mellitus and “unhealthy” body constitution presentations. *Journal of Integrative Medicine*.

[24] Leong P. K., Wong H. S., Chen J., Ko K. M. (2015). Yang/Qi invigoration: an herbal therapy for chronic fatigue syndrome with yang deficiency?. *Evidence-Based Complementary and Alternative Medicine*.

[25] van Dijk M., Dijk F. J., Bunschoten A. (2016). Improved muscle function and quality after diet intervention with leucine-enriched whey and antioxidants in antioxidant deficient aged mice. *Oncotarget *.

[26] Zhao L., Wu H., Qiu M. (2013). Metabolic signatures of kidney Yang deficiency syndrome and protective effects of two herbal extracts in rats using GC/TOF MS. *Evidence-Based Complementary and Alternative Medicine*.

[27] Kon R., Ikarashi N., Nagoya C. (2014). Rheinanthrone, a metabolite of sennoside A, triggers macrophage activation to decrease aquaporin-3 expression in the colon, causing the laxative effect of rhubarb extract. *Journal of Ethnopharmacology*.

[28] Bi D., Ning J., Xu Y. (2018). Effects of different doses of ginger-partitioned moxibustion on trefoil factor 1, mucin 5AC and epidermal growth factor receptor in rats with spleen deficiency syndrome. *Journal of Acupuncture and Tuina Science*.

[29] Zhang S. S., Li Q. G., Wei W., Lai Y. L. (2010). TCM consensus of management on irritable bowel syndrome. *China Journal of Traditional Chinese Medicine and Pharmacy*.

[30] Li X. J., Bai X. H., Chen J. X. (2014). Development and prospect of establishment method of traditional Chinese medicine animal models. *China Journal of Traditional Chinese Medicine and Pharmacy*.

[31] Zhu J. J., Su X. L., Liu S., Zhong S., Yao Y., Zhi Z. (2016). *Discussion on the establishment of rat model of diarrhea-predominant irritable bowel syndrome with yang deficiency in spleen and kidney based on the combination of disease and syndrome*.

