# Supplementary material for: A New Model of Diarrhea with Spleen-Kidney Yang Deficiency Syndrome
Source: Evid Based Complement Alternat Med. 2018 Sep 30;2018:4280343. doi: 10.1155/2018/4280343 (PMC6186382; doi:10.1155/2018/4280343)
Supplement: Supplementary Materials — Supplementary Material Supplementary Table 1: body weight of the four groups. Supplementary Table 2: food consumption and water intake of the four groups. Supplementary Table 3: diarrhea of the four groups. Supplementary Table 4: physical activities of the four groups. Supplementary Table 5: hormone level and serum D-xylose content of the four groups. [file 4280343.f1.pdf]

# Supplementary materials

Table S1. Body weight of the four groups.

| Group | N | 0w | 1w | 2w | 3w | 4w |
|---|---|---|---|---|---|---|
| C | 6 | 73.7±6.7 | 137.2±11.6 | 186.0±13.4 | 250.9±9.9 | 303.8±13.4 |
| H | 6 | 72.9±4.2 | 133.2±10.9 | 157.6±12.7[ab] | 209.2±20.8[ef] | 235.0±19.0[hij] |
| M | 6 | 78.2±10.8 | 129.0±7.5 | 161.9±8.8[cd] | 223.8±14.8[g] | 256.2±9.0[kl] |
| L | 6 | 76.3±8.6 | 135.8±10.7 | 176.5±12.2 | 238.1±11.0 | 273.8±10.8[m] |

[a] P=0.001, [c] P=0.002, [e] P=0, [g] P=0.005, [h] P=0, [k] P=0, [m] P=0.001, compared with group C. [b] P=0.013, [d] P=0.047, [f] P=0.003, [i] P=0, [l] P=0.037,, compared with group L. [j] P=, 0.014 , compared with group M.

Table S2. Food consumption and Water intake of the four groups.

| Group | N | Food consumption (g) | Water intake (ml) |
|---|---|---|---|
| C | 6 | 26.51±1.09 | 34.08±2.633 |
| H | 6 | 21.79±1.55[a] | 64.76±3.29[f] |
| M | 6 | 23.48±1.49[bc] | 57.02±3.53[ghi] |
| L | 6 | 24.29±1.56[de] | 44.88±2.86[jk] |

[b] P=0.037, [d] P=0.003, [g, j] P=0, compared with group H. [a] P=0, [c] P=0.001, [e] P=0.008, [f, h, k] P=0▵P＜ 0.01, compared with group C. [i] P=0, compared with group L.

Table S3. Diarrhea of the four groups.

| Group | N | Bristol score | | Fecal grains | |
|---|---|---|---|---|---|
| | | 0w | 4w | 0w | 4w |
| C | 6 | 3.42±0.11 | 3.92±0.28 | 4.83±1.17 | 5.83±1.47 |
| H | 6 | 3.44±0.14 | 5.89±0.23[abc] | 4.67±1.21 | 26.83±2.14[ghi] |
| M | 6 | 3.40±0.13 | 5.34±0.33[de] | 4.33±1.03 | 22.50±3.27[jk] |
| L | 6 | 3.37±0.07 | 4.59±0.36[f] | 4.50±1.05 | 16.17±4.47[l] |

[a, d, f, g, j, l] P=0, compared with group C. [b, h, k] P=0, [e] P=0.002, compared with group L. [c] P=0.005, [i] P=0.023, compared with group M.

Table S4. Physical activities of the four groups.

| Group | N | WLFST (s) | | FGST (g) | |
|---|---|---|---|---|---|
| | | 0w | 4w | 0w | 4w |
| C | 6 | 258.37±85.98 | 651.83±100.19 | 494.67±52.23 | 1507.92±103.94 |
| H | 6 | 269.13±70.83 | 448.66±82.67[ab] | 506.89±63.49 | 1244.53±113.53[de] |
| M | 6 | 253.35±78.80 | 528.27±94.52[c] | 505.57±67.48 | 1362.76±114.66[f] |
| L | 6 | 251.98±83.81 | 570.75±105.12 | 513.67±47.54 | 1462.17±102.27 |

[a] P=0.002, [c] P=0.037, [d] P=0, [f] P=0.032, compared with group C. [b] P=0.039, [e] P=0.002, compared with group L. WLFST, weight-loaded forced swimming test. FGST, forelimb grip strength test.

Table S5. Hormone level and serum D-xylose content

| Group | N | ACTH(pg/ml) | Cortisone(ng/ml) | 17-OHCS(ng/ml) | D-xylose(mmol/L) |
|---|---|---|---|---|---|

| | | | | | |
|---|---|---|---|---|---|
| C | 6 | 78.03±12.39 | 46.21±8.83 | 2.18±0.91 | 4.70±0.81 |
| H | 6 | 37.97±10.21[ab] | 29.34±6.79[de] | 0.83±0.38[fg] | 2.95±0.75[hi] |
| M | 6 | 51.91±10.59[c] | 36.80±9.00 | 1.49±0.55 | 3.47±0.71[j] |
| L | 6 | 66.34±16.61 | 42.65±9.84 | 1.61±0.61 | 3.97±0.82 |

[a] P=0, [c] P=0.002, [d] P=0.003, [f] P=0.002, [h] P=0.001, [j] P=0.012, compared with group C., [b] P=0.001, [e] P=0.016, [g] P=0.047, [i] P=0.033, compared with group L. ACTH, Adrenocorticotropic hormone. 17-OHCS, 17-hydroxycorticosteroids.
